# The regional decline and rise of tick-borne encephalitis incidence do not correlate with Lyme borreliosis, Austria, 2005 to 2018

**DOI:** 10.2807/1560-7917.ES.2021.26.35.2002108

**Published:** 2021-09-02

**Authors:** Karin Stiasny, Isabel Santonja, Heidemarie Holzmann, Astrid Essl, Gerold Stanek, Michael Kundi, Franz X Heinz

**Affiliations:** 1 Center for Virology, Medical University of Vienna, Vienna, Austria; 2 GfK Austria Healthcare, Vienna, Austria; 3 Astrid Eßl Consulting-Gesundheitsforschung, Wiener Neustadt, Austria; 4 Institute for Hygiene and Applied Immunology, Center for Pathophysiology, Infectiology and Immunology, Medical University of Vienna, Vienna, Austria; 5 Center for Public Health, Medical University of Vienna, Vienna, Austria

**Keywords:** tick-borne encephalitis (TBE), TBE virus, TBE epidemiology

## Abstract

**Background:**

Tick-borne encephalitis (TBE) virus is a human pathogen that is expanding its endemic zones in Europe, emerging in previously unaffected regions. In Austria, increasing incidence in alpine regions in the west has been countered by a decline in traditional endemic areas to the east of the country.

**Aim:**

To shed light on the cause of this disparity, we compared the temporal changes of human TBE incidences in all federal provinces of Austria with those of Lyme borreliosis (LB), which has the same tick vector and rodent reservoir.

**Methods:**

This comparative analysis was based on the surveillance of hospitalised TBE cases by the National Reference Center for TBE and on the analysis of hospitalised LB cases from hospital discharge records across all of Austria from 2005 to 2018.

**Results:**

The incidences of the two diseases and their annual fluctuations were not geographically concordant. Neither the decline in TBE in the eastern lowlands nor the increase in western alpine regions is paralleled by similar changes in the incidence of LB.

**Conclusion:**

The discrepancy between changes in incidence of TBE and LB support the contributions of virus-specific factors beyond the mere availability of tick vectors and/or human outdoor activity, which are a prerequisite for the transmission of both diseases. A better understanding of parameters controlling human pathogenicity and the maintenance of TBE virus in its natural vector−host cycle will generate further insights into the focal nature of TBE and can potentially improve forecasts of TBE risk on smaller regional scales.

## Introduction

Tick-borne encephalitis (TBE) is a viral disease that occurs in many parts of Europe and northern Asia [1]. According to the World Health Organization (WHO), ca 10,000 to 12,000 clinical cases of TBE are recorded each year, but the true incidence may be higher because of under-reporting in certain affected regions [1]. The disease is caused by the TBE virus, a flavivirus closely related to mosquito-transmitted human pathogens such as yellow fever, dengue, Zika and West Nile viruses [2]. There are three major genetic lineages of TBE virus, designated European, Siberian and Far-Eastern subtypes according to their principal geographic distribution [3]. In some areas, however, intermixing of these subtypes has occurred and two more subtypes (Baikalian and Himalayan) have recently been proposed [3]. Disease in infected humans can be effectively prevented by vaccination with inactivated vaccines [4]. There is evidence of a high degree of cross-protection among the different subtypes [5,6].

In its natural cycle, the TBE virus is transmitted from infected hard ticks (*Ixodes ricinus* - primarily in Europe and *Ixodes persulcatus* - primarily in Asia) to small mammals that serve as reservoir hosts [7]. Infection of uninfected ticks can occur when they feed on infected hosts during viraemia or through simultaneous feeding with an infected tick in close proximity on the same host animal, by a process called non-viraemic transmission [8]. The relative roles of these different modes of tick infection in the natural TBE virus transmission cycles are still under debate [7]. Humans are incidental and dead-end hosts only and do not play any role in the maintenance of the virus in nature [7].

A number of reports provide evidence for the expansion of TBE-endemic areas in Europe in recent years [9]. Increases have been noted in the north of Europe, affecting Scandinavian countries [10-13] as well as the northern part of European Russia [14]. In addition, countries in the west, considered free of TBE for decades, have reported first isolations of TBE virus from locally collected ticks and/or first detections of autochthonous cases in humans. These countries include the Netherlands [15] and the United Kingdom [16]. The acquisition and persistence of new natural foci of TBE virus replication is usually attributed to the long-range transportation of infected ticks (e.g. through birds or other animals [7]) and climatic changes that favour the establishment of tick habitats and natural cycles of TBE virus in previously unaffected zones [14,17].

Based on modelling studies, Randolph and Rogers predicted a potentially countercurrent trend imposed by climate change, i.e. the contraction and/or complete disappearance of extant natural foci of TBE virus in certain areas due to an increase in temperature and decrease in moisture [18]. A possible explanation for such a contraction could be the fragility of the natural transmission cycle of TBE virus (including the requirement for temporal synchronicity of nymphal and larval development to allow co-feeding transmission [8]) that may be disrupted by climate change. We indeed observed indications of such opposing trends in central Europe, i.e. concomitant increasing and decreasing incidence of TBE in humans in different endemic areas within Austria on a small geographical scale, that cannot be ascribed to vaccination [19]. It is difficult to establish explanations for these countercurrent trends because of the complexity of factors that can affect transmission and the incidence of TBE in humans. These not only include climatic factors and seasonal patterns that control the life cycle of ticks and their mammalian hosts, but also weather conditions and social habits of humans that influence their risk of exposure to infected ticks [20,21]. In certain regions, for example in the Baltic states, epidemiological changes have been primarily attributed to non-biological causes, such as political and sociological changes [22-43].

Since Lyme borreliosis (LB) is transmitted by the same vector (*Ixodes ricinus*) as TBE and is maintained in the same rodent reservoir [23], one would expect TBE incidence to be mirrored by changes in LB, if these were solely dependent on tick populations and/or climate changes. In both instances only a small proportion of infected individuals require hospitalisation [26,27]. In our study, we therefore compared the declines and increases of hospitalised cases of TBE in different regions of Austria from 2005 to 2018 with the incidences of hospitalised cases of LB.

## Methods

### Documentation of tick-borne encephalitis cases

Tick-borne encephalitis has been a notifiable disease in Austria since 2012 [24]. TBE cases are documented by the Center for Virology at the Medical University of Vienna, which is the National Reference Laboratory (NRL) for TBE virus and other flavivirus infections. Only hospitalised patients with a serologically diagnosed infection with TBE virus are counted as cases. Confirmation is based on TBE virus IgM and IgG ELISA results performed by the NRL. The diagnostic algorithms and national awareness campaigns for TBE and other tick-borne diseases were not changed during the study period.

After confirmation, questionnaires for all hospitalised patients were sent by the NRL to the treating physicians in the hospitals, requesting information on tick bites and the possible geographical site of infection. From 2005 to 2018, the place of residence of TBE patients matched hospitalisation within the corresponding federal province in 97.5% of cases.

### Documentation of Lyme borreliosis cases

Information of hospitalised cases of LB was based on hospital discharge records for the years 2005 to 2018. These data contain all cases registered in a public hospital in Austria with the ICD code A69.2x (Lyme disease, including meningitis and other neurological disorders, arthritis and other conditions associated with Lyme disease). All individuals with a place of residence in Austria were included. The incidences in federal provinces were derived from the postal code of patient’s home address.

### Mapping of infection sites

Probable TBE virus infection sites were geocoded and processed for spatial mapping with quantum geographic information system (QGIS) (https://www.qgis.org/). Spatially close sites were aggregated using a 2 km grid for Austria, and centroids were calculated for each square. The centroids formed the centre of circles with diameters proportional to the number of documented sites within this area. The base map was generated with open access data from Statistik Austria (borders of Austria and its federal countries, https://www.statistik.at/web_de/statistiken/index.html), Natural Earth Data (rivers, lakes, cities, http://www.naturalearthdata.com) and Global Multi-Resolution Topography (GMRT) synthesis data of the Marine Geoscience Data System (MGDS) [25] (http://www.marine-geo.org/tools/GMRTMapTool).

### Calculation of incidence rates

**Figure 1:**
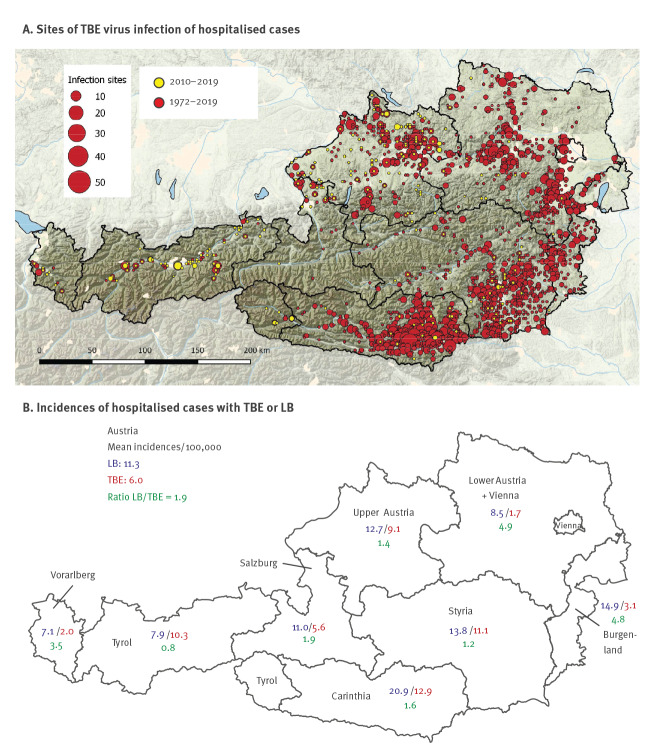
Probable sites of TBE virus infection of hospitalised cases (1972–2019) and mean incidences of TBE (n = 970 cases) and LB (n = 14,055 cases), Austria 2005−2018

LB: Lyme borreliosis; TBE: tick-borne encephalitis.

The overall incidence rate for LB patients and unvaccinated TBE patients were calculated nationally, and for each individual federal province (Vienna was included in the counts of Lower Austria because of its location; Figure 1B).

Panel A shows sites of TBE virus infection of hospitalised cases. Red filled circles: cumulative infection sites from 1972 to 2019. Yellow circles: cumulative infection sites recorded in the last 10 years (from 2010 to 2019).

Panel B shows incidences (geometric means) of cases hospitalised with TBE (red) or LB (blue) between 2005 and 2018 in Austria and its individual federal provinces. The ratios of these incidences (LB/TBE) are indicated in green italics.

Incidence: number of cases per 100,000 population.

Population data were obtained from Statistik Austria and data on TBE vaccination coverage in each federal state were obtained through postal surveys conducted by Growth from Knowledge (GfK) Austria GmbH (https://www.gfk.com/de/home), an institute for market and opinion research in Vienna [28]. Based on previous studies [28,29], TBE incidence among unvaccinated persons was calculated using the actual number of cases that occurred among unvaccinated persons from 2005 to 2018. The assignment of cases to individual federal provinces was based on the hospitalisation site.

Geometric mean incidences from 2005 to 2018 (Figure 1B) were calculated from annual incidences during this period. In some federal provinces with no recorded TBE cases (Burgenland, Vorarlberg, Supplementary Table S1), geometric mean of TBE incidences were computed by weighting so that the annual population size replaced 0 with half the expectancies from cubic spline fits.

### Ethical statement

This study was based on aggregated surveillance data and ethical approval was not required.

## Results

The analysis of probable infection sites in Austria confirmed a previously observed trend, i.e. the establishment of new endemic foci in alpine regions in the west, and almost complete disappearance of infection sites in regions in the east (Figure 1A). These eastern regions comprised some of the most heavily affected endemic areas in Austria until the end of the 1980s [19].

**Figure 2:**
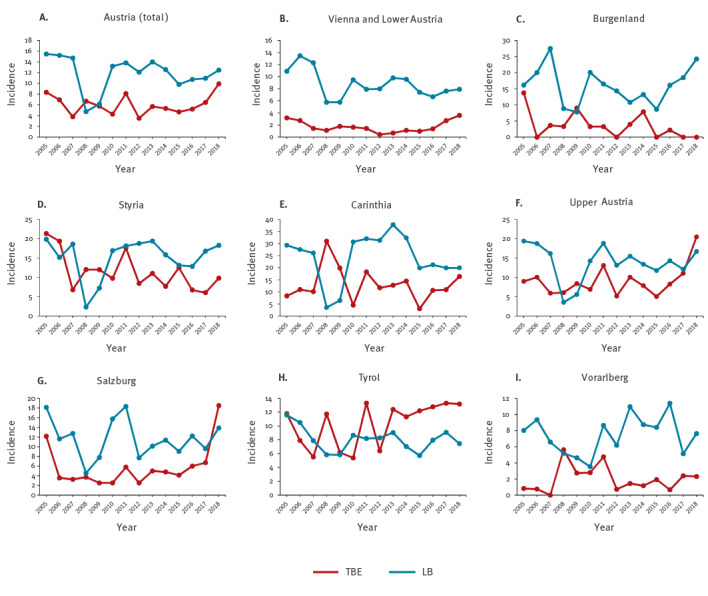
Annual incidences of patients hospitalised with TBE (n = 970 cases) and LB (n = 14,055 cases) by year and federal provinces, Austria, 2005−2018

LB: Lyme borreliosis; TBE: tick-borne encephalitis.

In the next step, we compared the incidences of hospitalised cases of TBE and LB between 2005 and 2018. Since the incidence of TBE in Austria has declined strongly thanks to vaccination [28,29], we calculated it for the unvaccinated population only to see epidemiological changes independent of vaccination. Results of these comparative analyses are presented on a national level and regional level, i.e. for each federal province (Figures 1B and 2; Supplementary Tables S1 and S2).

Incidence: number of cases per 100,000 population.

The mean incidence of hospitalised LB cases country-wide during the study period was about twice as high as that of TBE (11.3 vs 6.0). However, this ratio varied strongly in different federal provinces, with the lowest incidence of 0.8 in Tyrol and highest incidence of 4.9 in Vienna/Lower Austria (Figure 1B). Independent of this geographical disparity, the incidences of both TBE and LB were subject to strong annual variations, consistent with the complexities of transmission dynamics (Figure 2). These variations, however, do not occur in parallel for the two diseases in the same regions. We highlight the years 2008 and 2009, which had disproportionately low LB incidences across all federal provinces (Figure 2), but peak TBE incidences in Carinthia (Figure 2E) and Tyrol (Figure 2H) as the most striking examples of this incongruence. We therefore conclude that the incidences of the two tick-transmitted diseases are discordant not only on a small geographical scale within Austria but also with respect to their variation from season to season.

**Figure 3:**
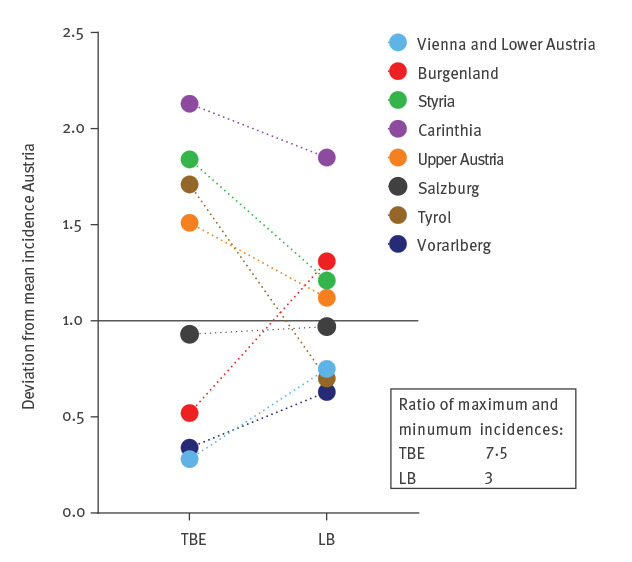
Deviations of the mean incidences of TBE and LB in individual federal provinces from the mean of the whole country, Austria, 2005−2018

LB: Lyme borreliosis; TBE: tick-borne encephalitis. Variation of mean incidences (geometric means) is expressed as the fold difference from the mean incidence in Austria. Dashed lines connect the dots of the homologous federal provinces in the two panels. Incidence: number of cases per 100,000 population.

We further analysed the extent of variation of incidences among individual federal provinces and quantified their deviation from the mean incidence in the whole country. Figure 3 shows that regional differences (as reflected by the variation of incidences in individual federal provinces) were substantially more pronounced for TBE than for LB, with maximum/minimum factors of 7.5 and 3, respectively. The most contrasting examples were the Burgenland in the east (red dots in the figure) and Tyrol in the west. The average incidence of TBE over the whole observation period in the Burgenland was well below the Austrian mean, whereas that of LB was above the mean (Figure 3). Most strikingly, there were no cases of TBE in the Burgenland in the years 2017 and 2018, contrasting to a high incidence of LB in the same years (Figure 2C). Tyrol (brown dots in Figure 3) showed the opposite trend and is the only province in Austria in which the TBE incidence has consistently superseded that of LB since 2013 (Figure 1B and 2H).

## Discussion

The observed shifts in TBE virus epidemiology may be the result of several mutually interacting factors including climate change, environmental change, abundance of ticks and their hosts, economic fluctuations and social habits [17,22,30]. Our analyses suggest that factors other than those directly influencing propagation and abundance of ticks in their habitats or changes in human behaviours leading to tick exposure are responsible for the geographical and temporal discrepancies between the incidences of TBE and LB.

We found countercurrent trends for these two pathogens both geographically and on a temporal scale, although they are transmitted by the same vector and depend on the same natural host system [23]. One of the most apparent differences observed was the substantial decline of TBE in the east of the country (federal province of Burgenland), where the incidence of LB was disproportionately high. These data indicate that either less pathogenic virus mutants had replaced the original wildtype, or the conditions for maintaining TBE virus in its natural ecological cycle became unfavourable, despite the maintenance of the vector and its hosts as indicated by the unchanged high number of hospitalised LB patients. The infection rate of *I. ricinus* ticks with *Borrelia* in Austria appears to be constant over the years (ca 26%). The most frequently detected *Borrelia* genospecies are *Borrelia afzelii* (ca 56%), *B. burgdorferi sensu stricto* (ca 27%), *B. valaisiana* (ca 25%), and *B. garinii* (ca 20%) [31]. The most prominent pathogenic *Borrelia* genospecies is *B. afzelii* in skin manifestations (ca 90%), *B. garinii* predominates in Lyme neuroborreliosis (ca 67%) [32]. It is unlikely that the disparities between TBE and LB cases are related to differences in access to hospitals because these would affect both diseases equally, and there are high standards of medical care throughout Austria.

A possible explanation for the discrepancies observed would be the proposed dependency of TBE virus on so-called non-viraemic co-feeding transmission, which refers to the transmission of TBE virus from infected nymphs to uninfected larvae feeding in close proximity on the same rodent host [8]. This mechanism of enzootic transmission requires sufficient coincidence between larval and nymphal development. Less temporal overlap of the two developmental stages can be caused by climatic changes [20], resulting in suboptimal conditions for viral transmission and ultimately the loss of the virus from its transmission cycle. Such a scenario was indeed proposed by Randolph and Rogers in their theory of the effects of climate change on the epidemiology of TBE [18]. Interestingly, the incidence of TBE in Hungary has also strongly declined since 1996 and the trend appears to continue at least until 2015 [33,34]. This country is adjacent to the eastern border of Austria and the Burgenland and has a similar Pannonian climate that differs substantially from the alpine regions of Austria and Switzerland where an upsurge of TBE was recorded [19,35].

Additional factors can be hypothesised to be responsible for the discordant epidemiology of TBE and LB, or may act in concert with non-viraemic transmission of TBE virus. The infectious cycle of TBE virus in ticks involves replication in cells lining the midgut, dissemination to the haemolymph, and subsequent infection of cells of different tissues to reach the highest titres in the ticks’ salivary glands [36]. In each cell, the viral life cycle involves a plethora of virus-specific and cell-dependent processes that may be affected by environmental conditions (especially temperature variations), ultimately influencing the dynamics and persistence of the virus in its vector [37]. In addition, the balance of virus replication and its counteraction by processes of innate immunity in the tick [38] might be modulated by specific temperature conditions to the advantage or disadvantage of the virus. Together, these factors define the optimal temperatures for transmission, which may be negatively affected when the system falls below a certain threshold of efficiency.

In addition to direct temperature effects on virus-host interactions, the microbiome in the midgut of the vector may exert important influences on pathogen replication and transmission [21]. Studies have shown that pathogens (including TBE virus and *Borrelia*) can have specific effects on tick behaviour (e.g. mobility) [39] and affect physiological functions such as apoptosis, innate responses and tick fitness in general [21]. It is currently unclear whether the microbiomes of ticks across geographical regions differ. Such differences can potentially affect the balance between innate antiviral immunity and antagonising viral factors that would be required for optimal transmission [40]. Since imbalances would be pathogen-specific, they might contribute to the region-specific upsurges and declines in TBE incidence as well as the discordance with LB incidence observed in our study. Variations in the tick microbiome might also play a role in the characteristic focal occurrence of TBE virus in its endemic areas.

Our findings are relevant for studies that attempt to forecast incidence of TBE [33,41]. The problem is exemplified in Hungary, where forecasting data suggested a rapid increase beginning around 2010 [33], whereas observed incidences have continuously declined during the same time window [33,34]. This result was unexpected since the algorithm applied in the study took into account changes in non-viraemic transmission between co-feeding ticks. Whether the discrepancy in forecasts and actual numbers can be ascribed to TBE underdiagnoses and/or under-reporting and/or vaccination [33], will require further analyses of these parameters over time. Forecasts of TBE for the whole of Austria (as well as Germany and Switzerland) for the years 2019–2021 used the fructification index of the European beech during the previous 2 years as the most important predictor of the TBE virus transmission cycle [41]. Good matches were found between predicted and recorded cases in 2019 (82 +/− 12 vs 108) and 2020 (156 +/− 19 vs 216) [41,42,44]. However, the overall TBE incidence in Austria is a composite of increasing incidences in the west and decreasing incidences in the east [19], apparently controlled by opposing factors not equally distributed over the whole country. It will be interesting to see whether forecasting methods can be adjusted to account for the strikingly different developments observed on a small regional scale.

Our study has several limitations. Specifically, we did not study environmental changes that might affect tick habitats and abundance as well as human risk behaviour. We also did not assess pathogen factors that contribute to severe disease requiring hospitalisation.

Although we believe it unlikely, we cannot exclude that TBE virus strains circulating in the east have acquired mutations that reduce transmission or pathogenicity for humans. In any case, the decline in TBE in the eastern part of the country despite a continuing high prevalence of LB indicates that other factors beyond the prevalence of ticks and human exposure, which are common to both pathogens, underlie the epidemiological phenomena observed.

## Conclusions

Our study points to ongoing changes in virus-specific factors of TBE that control the circulation and maintenance of TBE virus in its natural cycle and/or affect disease in humans. These factors might be responsible for the upsurge in TBE hospitalisations in newly endemic areas and declining hospitalisations or disappearance of TBE in regions with a history of high disease incidence.

Since the changing patterns observed for hospitalised cases of TBE do not mirror those seen for LB, it is likely that parameters beyond those influencing tick abundance in general and/or human exposure to tick bites are responsible.

Future studies to elucidate the unknown phenomena underlying the pathogenicity, ecological cycle and transmission efficiency of TBE virus may help to improve methods of disease forecasting and to resolve the conundrum of TBE focality and divergent developments on a small geographical scale.

## Supplementary Data

787000 Supplement
