# Supplementary material for: The regional decline and rise of tick-borne encephalitis incidence do not correlate with Lyme borreliosis, Austria, 2005 to 2018
Source: Euro Surveill. 2021 Sep 2;26(35):2002108. doi: 10.2807/1560-7917.ES.2021.26.35.2002108 (PMC8414957; doi:10.2807/1560-7917.ES.2021.26.35.2002108)
Supplement: Supplement [file 20-02108_STIASNY_Supplement.pdf]

*Disclaimer: This supplementary material is hosted by Eurosurveillance as supporting information alongside the article "**The regional decline and rise of tick-borne encephalitis incidence do not correlate with Lyme borreliosis, Austria, 2005 to 2018"** on behalf of the authors, who remain responsible for the accuracy and appropriateness of the content. The same standards for ethics, copyright, attributions and permissions as for the article apply. Supplements are not edited by Eurosurveillance and the journal is not responsible for the maintenance of any links or email addresses provided therein.*

Supplementary Table 1:
Incidence of TBE/100,000 in Austria and its federal provinces, 2005-2018

Year	Austria (total)	Vienna + Lower Austria	Burgen-land	Styria	Carinthia	Upper Austria	Salzburg	Tyrol	Vorarl-berg
2005	8.33	3.20	13.75	21.34	8.31	8.99	12.16	11.80	0.82
2006	6.95	2.75	0.00	19.35	10.96	10.06	3.56	7.90	0.75
2007	3.79	1.45	3.65	6.80	10.13	5.93	3.25	5.52	0.00
2008	6.70	1.12	3.31	12.03	30.99	6.08	3.71	11.73	5.62
2009	5.77	1.78	9.03	12.00	19.89	8.42	2.53	6.17	2.73
2010	4.29	1.65	3.27	9.77	4.57	6.91	2.53	5.38	2.79
2011	8.11	1.44	3.26	17.66	18.30	13.08	5.79	13.31	4.75
2012	3.52	0.42	0.00	8.46	11.71	5.18	2.52	6.41	0.73
2013	5.71	0.68	3.97	11.04	12.76	10.05	5.00	12.40	1.45
2014	5.34	1.13	7.88	7.70	14.46	7.85	4.78	11.34	1.16
2015	4.69	0.99	0.00	12.54	3.05	5.08	4.12	12.19	1.93
2016	5.22	1.37	2.20	6.74	10.64	8.25	6.00	12.77	0.66
2017	6.45	2.73	0.00	6.07	10.93	11.07	6.71	13.31	2.40
2018	9.93	3.59	0.00	9.81	16.39	20.46	18.52	13.19	2.31

Supplementary Table 2:

Incidence of LB/100,000 in Austria and its federal provinces, 2005-2018

Year	Austria (total)	Vienna + Lower Austria	Burgen-land	Styria	Carinthia	Upper Austria	Salzburg	Tyrol	Vorarl-berg
2005	15.48	10.91	16.16	19.86	29.33	19.39	18.15	11.58	8.02
2006	15.23	13.45	20.03	15.15	27.53	18.76	11.61	10.50	9.36
2007	14.72	12.31	27.45	18.62	26.08	16.16	12.74	7.88	6.58
2008	4.76	5.77	8.87	2.33	3.57	3.55	4.56	5.85	5.19
2009	6.15	5.78	7.77	7.22	6.44	5.61	7.79	5.83	4.62
2010	13.19	9.48	20.07	16.92	30.67	14.26	15.75	8.65	3.52
2011	13.83	7.92	16.49	18.14	31.99	18.83	18.35	8.19	8.65
2012	12.08	7.99	14.33	18.77	31.31	13.13	7.73	8.28	6.19
2013	14.00	9.81	10.80	19.38	37.80	15.47	10.13	9.05	10.97
2014	12.58	9.58	13.20	15.85	32.34	13.41	11.37	7.04	8.75
2015	9.80	7.40	8.64	13.14	19.87	11.77	9.05	5.73	8.40
2016	10.74	6.69	16.11	12.87	21.21	14.31	12.23	7.95	11.38
2017	10.95	7.62	18.48	16.80	19.97	12.12	9.62	9.09	5.12
2018	12.47	7.91	24.23	18.29	19.97	16.71	13.90	7.44	7.63
